# Syk-dependent alternative homologous recombination activation promotes cancer resistance to DNA targeted therapy

**DOI:** 10.21203/rs.3.rs-2922520/v1

**Published:** 2023-06-09

**Authors:** Qin Zhou, Xinyi Tu, Xiaonan Hou, Jia Yu, Fei Zhao, Jinzhou Huang, Jake Kloeber, Anna Olson, Ming Gao, Kuntian Luo, Shouhai Zhu, Zheming Wu, Yong Zhang, Chenyu Sun, Xiangyu Zeng, Kenneth Schoolmeester, John Weroha, Liewei Wang, Robert Mutter, Zhenkun Lou

**Affiliations:** Department of Radiation Oncology, Mayo Clinic; Department of Radiation Oncology, Mayo Clinic; Department of Oncology, Mayo Clinic; Department of Molecular Pharmacology and Experimental Therapeutics, Mayo Clinic; Department of Oncology, Mayo Clinic; Department of Oncology, Mayo Clinic; Department of Oncology, Mayo Clinic; Department of Oncology, Mayo Clinic; Research Center for Eco-Environmental Sciences, Chinese Academy of Sciences; Department of Oncology, Mayo Clinic; Department of Oncology, Mayo Clinic; Department of Oncology, Mayo Clinic; Department of Oncology, Mayo Clinic; AMITA health saint joseph hospital; Department of Oncology, Mayo Clinic; Laboratory Medicine and Pathology, Mayo Clinic; Department of Oncology, Mayo Clinic; Department of Molecular Pharmacology and Experimental Therapeutics, Mayo Clinic; Department of Radiation Oncology, Mayo Clinic; Department of Oncology, Mayo Clinic

## Abstract

Enhanced DNA repair is an important mechanism of inherent and acquired resistance to DNA targeted therapies, including poly ADP ribose polymerase inhibition. Spleen associated tyrosine kinase (Syk) is a non-receptor tyrosine kinase known to regulate immune cell function, cell adhesion, and vascular development. Here, we report that Syk can be expressed in high grade serous ovarian cancer and triple negative breast cancers and promotes DNA double strand break resection, homologous recombination (HR) and therapeutic resistance. We found that Syk is activated by ATM following DNA damage and is recruited to DNA double strand breaks by NBS1. Once at the break site, Syk phosphorylates CtIP, a key mediator of resection and HR, at Thr-847 to promote repair activity, specifically in Syk expressing cancer cells. Syk inhibition or genetic deletion abolished CtIP Thr-847 phosphorylation and overcame the resistant phenotype. Collectively, our findings suggest that Syk drives therapeutic resistance by promoting DNA resection and HR through a novel ATM-Syk-CtIP pathway, and that Syk is a new tumor-specific target to sensitize Syk-expressing tumors to PARPi and other DNA targeted therapy.

## Introduction

Cells employ a series of repair mechanisms to maintain genome stability following endogenous or exogenous damage to one or both strands of DNA^1^. The DNA double strand break (DSB) is the most lethal DNA lesion encountered by cells and must be repaired for cell survival. In G0 and G1, DNA double strand breaks (DSBs) are primarily repaired by nonhomologous end joining (NHEJ), a potentially error-prone DNA repair mechanism^2^. Although NHEJ is also available for repair in S and G2 phases, DSBs are preferentially repaired during these cell cycle phases by homologous recombination (HR), a relatively error-free mechanism which requires an intact sister chromatid to serve as a template to carry out repair with fidelity^3^. For DSB repair to proceed by HR over NHEJ there must be DSB resection, where 3’ single strand DNA overhangs are generated to serve as a platform for HR initiation^4,5^. The human C-terminal binding protein interacting protein (CtIP) protein is an endonuclease that interacts with its partner, the MRE11–RAD50–NBS1 (MRN) complex, to promote DSB resection^6–8^. NBS1 and BRCA1, another key factor in HR, are known binding proteins of CtIP^11,12^. Thus, CtIP is a key regulator of DNA DSB repair by HR.

Cells require intact HR to repair damage induced by multiple DNA damaging therapies including cisplatin and poly ADP ribose (PARP) inhibitors. Thus, tumors with deficiencies in HR, such as are commonly observed in subtypes of breast and ovarian cancer, are highly sensitive these agents, whereas robust HR repair is a potential mechanism of inherent and acquired therapeutic resistance^9–12^. There is a pressing need for new tumor-specific strategies to overcome resistance to PARP inhibition (PARPi), platinum and other DNA damaging therapies^13^.

Spleen associated tyrosine kinase (Syk) is a non-receptor tyrosine kinase which mediates signal transduction downstream of a variety of transmembrane receptors, including classical immunoreceptors such as the B-cell receptor (BCR). Syk is best known for its role in the regulation of several biological processes including innate and adaptive immunity, cell adhesion, osteoclast maturation, platelet activation and vascular development^14^. However, Syk has also been reported to have both tumor promoter and tumor suppressor functions in cancer^15–17^. For example, Syk promotes cell survival in most BCR hematopoietic malignancies^15^, but a reduction in Syk expression and activity through alternative processing of Syk mRNA promotes malignant progression of CD19 + CD10- pro-B cell acute lymphoblastic lymphoma^18^. In addition, Syk expression in pre-treatment ovarian cancer specimens has previously been associated with ovarian cancer cell invasiveness by mediating actin filament assembly and dynamics and microtubule-associated proteins through phosphorylation of cortactin, cofilin, and tubulins^19^. In contrast, Syk loss has been associated with a more malignant breast cancer phenotype^20,21^

In this study, we have discovered that Syk can be overexpressed in high-grade ovarian cancer (HGSOC), and estrogen receptor (ER) negative, progesterone receptor (PR) negative, and human epidermal growth factor receptor 2 (HER2) gene negative breast cancer (triple negative breast cancer, [TNBC]) and promotes HR and resistance to DNA targeted therapy. We have found that Syk can be activated and recruited to DNA DSBs in an ATM dependent manner in Syk expressing tumor cells, where Syk phosphorylates CtIP and promotes CtIP-mediated end-resection and HR. Enhanced Syk-mediated DSB resection and HR increased resistance to PARPi and cisplatin specifically in Syk expressing tumor cells, and could be overcome by Syk inhibition. Collectively, our findings suggest that targeting Syk and the novel ATM-Syk-CtIP pathway is a promising strategy to overcome therapeutic resistance resulting from proficient HR repair.

## RESULTS

### Syk is overexpressed in HGSOC and TNBC and is associated with resistance to DNA targeting therapy

**Figure 1:**
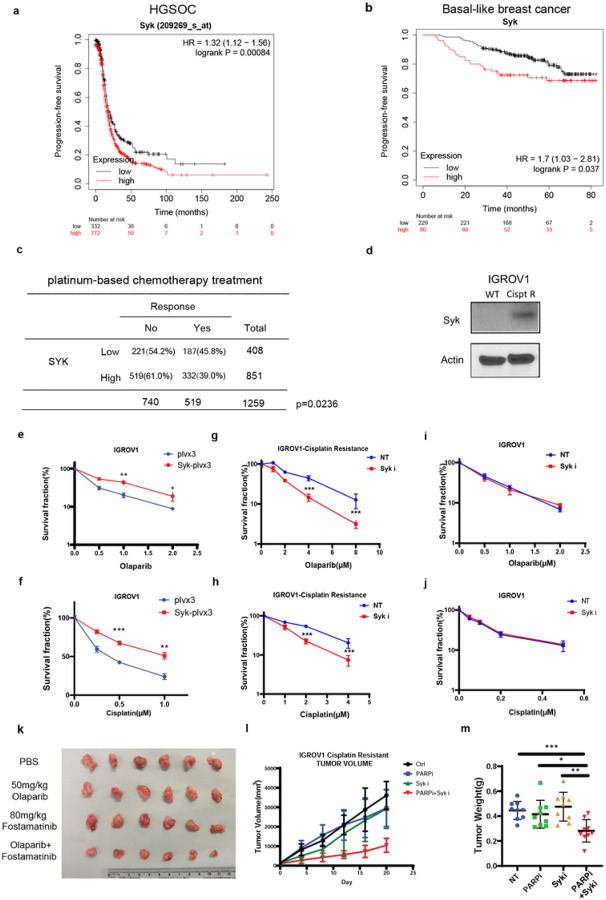
Syk expression is associated with resistance to DNA damaging therapy (a-b) Kaplan-Meier progression-free survival curves of patients with HGSOC (a) and PAM-50 basal-like breast cancer subtype (b) separated by Syk expression levels in TCGA. (c) Association between clinical responses to platinum-based chemotherapy and Syk expression in HGSOC in TCGA. (d) Syk expression is assessed by immunoblot in the indicated wild-type (WT) and cisplatin (Cispt) or PARP inhibitor (PARPi) resistant (R) HGSOC subline IGROV1 cells. (e,f) IGROV1 cells were infected with lentivirus expressing the indicated plasmids. Clonogenic survival was then assessed following exposure to olaparib (e) or cisplatin (f) at the indicated doses. (g-j) Clonogenic survival assay demonstrating sensitivity of cisplatin resistant IGROV1(g,h) and wild-type IGROV1 (i,j) sublines to combination therapy with Syk inhibitor (R406, 0.1μM) with either olaparib (g,i) or cisplatin (h,i) at the indicated doses. Error bars represent SEM from three independent experiments. Error bars represent SEM from three independent experiments. (k-m) IGROV1 cisplatin resistant cells were subcutaneously injected into the flank of NOD-SCID mice. Mice were treated with saline, Fostamatinib (80mg/kg gavage 6 times) with or without olaparib (50 mg/kg i.p. 10 times). Tumor images (k), tumor volume (l), and tumor weight (m) are shown. Data points represent mean ± SEM from n = 8 biologically independent samples. P values were obtained by two-sided unpaired t test. *p<0.05, **p<0.01,***p<0.001

Loss of components of DNA-maintenance machinery during tumor progression is a common feature of cancer and can render tumors susceptible to DNA targeted therapy^22^. However, these inherent defects may be compensated for by activation of alternative backup pathways, with the associated increase in DNA damage response and repair activity being an important mechanism of therapeutic resistance^23^. Interestingly, we observed that Syk RNA expression was associated with significantly worse progression-free survival (PFS) in HGSOC (Fig. 1a) and the basal subtype of breast cancer (Fig. 1b), but not the other common subtypes of ovarian and breast cancer (Extended Data Fig. 1a-d) in the TCGA database. HGSOC and basal-like breast cancer, the large majority of which are TNBC, share molecular features including high rates of inactivation of HR DSB repair by genetic and epigenetic mechanisms^24–26^. Of note, in patients with HGSOC high Syk RNA expression was associated with lower rates of response (Fig. 1c) and worse progression-free survival (Extended Data Fig. 1e) following cisplatin therapy in patients with HGSOC in TCGA. These results were consistent with a prior report demonstrating more highly expressed Syk in recurrent HGSOC specimens following platinum-based chemotherapy compared to primary untreated specimens^27^. We also observed robust Syk expression in a panel of platinum-resistant HGSOC patient-derived xenograft (PDX) models and TNBC PDXs established from chemotherapy resistant residual surgical specimens of early-stage TNBC patients in our laboratory (Extended Data Fig. 1g-h)^28^. Moreover, we found that Syk was overexpressed in cisplatin resistant and PARP inhibitor resistant sublines of HGSOC IGROV1 cells, (Fig. 1d). Since platinum and PARP inhibitors induce DNA damage, these results raised the possibility that Syk expression may be associated with resistance to DNA damaging therapy.

To examine the impact of Syk expression on response more specifically we overexpressed Syk in the parental non-Syk expressing IGROV1 HGSOC cell line and evaluated the sensitivity to cisplatin and PARP inhibition (Fig. 1e–f, Extended Data Fig. 1f). Syk expression rendered these cells more resistant to both cisplatin (Fig. 1e) and PARP inhibition (Fig. 1f). In contrast, knockdown of Syk or treatment with a specific small molecule Syk inhibitor, R406, could sensitize Syk-expressing cisplatin resistant IGROV1 and other Syk-expressing HGSOC and breast cancer cells to these agents (Fig. 1g–h and Extended Data Fig. 2a-d, 2h). Syk knockdown or Syk inhibition also sensitized Syk expressing cancer cells to (Extended Data Fig. 2e-g). In contrast, Syk inhibition did not sensitize Syk non-expressing IGROV1 cells to DNA damaging therapy (Fig. 1i–j). Moreover, the sensitivity of Syk non-expressing epithelial cell lines TERT-RPE and MCF10A to cisplatin, PARP inhibition, and radiation were minimally impacted by Syk inhibition (Extended Data Fig. 2i-m). These results suggested that targeting Syk could be a tumor specific approach to sensitize Syk-expressing tumor cells to DNA damaging therapy.

To further examine the potential of targeting Syk to improve the therapeutic ratio of DNA damaging therapy in Syk-expressing tumors, we also evaluated the impact of Syk inhibitor combination therapy *in vivo*. R406 is the active metabolite of Fostamatinib (R788), which is a clinically approved Syk inhibitor for the treatment of idiopathic thrombocytopenia^25^. We randomized Syk-expressing IGROV1 cisplatin resistant tumor xenografts to control, PARP inhibition, Fostamatinib, or the combination of PARP inhibition plus Fostamatinib. The xenografts were highly resistant to PARP or Syk inhibitor monotherapy. However, the combination of PARP inhibition and Syk inhibition significantly delayed tumor growth (Fig. 1k–m). A mechanistic link between Syk and the cellular response to DNA damage had not previously been defined. However, these results collectively suggested the possibility that Syk may play a role in promoting therapeutic resistance by upregulating DNA repair.

### Syk promotes DSB repair by HR

**Figure 2:**
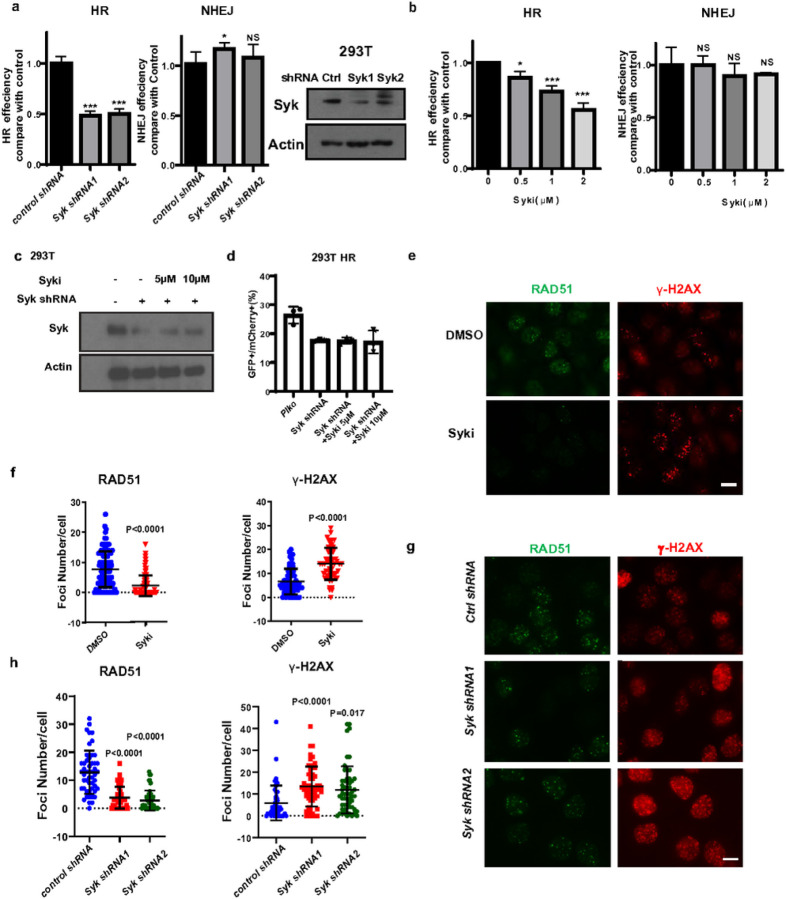
Syk is required for HR activity (a-b) GFP-tagged HR and NHEJ reporter plasmids were transfected into 293T cells and cells infected with lentiviruses expressing Syk shRNAs (a), or treated with the indicated doses of the Syk inhibitor, R406 (b) and repair efficiency was assessed using flow cytometry. (c-d) Endogenous Syk was knocked down using shRNA in 293T cells expresing the HR reporter plasmid as in (a). Control and Syk knockdown cells were then treated with or without R406 at the indicated doses after which immunoblot was performed on cell lysates using the indicated antibodies (c) and HR activity was assessed (d), as above. *p<0.05, **p<0.01,***p<0.001. (e-h) OVCAR7 cells were exposed to R406 (1 μM) or infected with lentivirus-expressing control (Ctrl) or Syk shRNAs and exposed to 4Gy irradiation. Cells were fixed and stained 4 hours after irradiation with RAD51(Green) and γ-H2AX(Red) antibodies. (e, g) Representative immunofluorescence images are shown. Scale bar for IF images, 20μm. (f, h) The number of RAD51 foci per cell and the number of γ-H2AX foci per cell after the indicated treatments were quantified. Representative data (mean±SEM) are shown from three independent experiments. n=50 cells. All P values above obtained by two-sided unpaired t test

Cellular proficiency of DSB repair by HR is an important determinant of sensitivity to platinum and PARP inhibition^3,23^. Therefore, we hypothesized that Syk may play a role in promoting HR in Syk expressing tumor cells. To investigate a potential role of Syk in DSB repair we first knocked down Syk in 293T cells and examined the impact of Syk on DSB repair by HR and NHEJ utilizing established reporter assays^29^. Syk knockdown downregulated the activity of HR, but not NHEJ (Fig. 2a). In addition, Syk inhibition reduced HR, but not NHEJ activity, in a dose dependent manner (Fig. 2b). However, Syk inhibition did not further reduce HR function in Syk knockdown cells, suggesting that Syk inhibition and Syk knockdown were disrupting HR specifically along the same pathway (Fig. 2c–d). Of note, backup DSB repair pathways alternative NHEJ (alt NHEJ) and single strand annealing (SSA) were not impacted by Syk depletion (Extended Data Fig. 3a,b), further suggesting that Syk may promote DSB repair and resistance to DSB inducing agents specifically through HR. To further examine the impact of Syk on DNA repair and HR, we knocked down Syk in Ovcar7 cells (Extended Data Fig. 2h) and assessed IR-induced γ-H2AX and RAD51 foci formation. γ-H2AX is a chromatin modification induced at DSBs immediately following DNA damage, and Rad51 recruitment to DSBs is required for repair to proceed by HR^30^. Both Syk knockdown or Syk inhibition reduced RAD51 recruitment to DSB sites and resulted in greater number of unresolved γ-H2AX foci following IR (Fig. 2e–h). We concluded from this series of experiments that Syk promotes more efficient DSB repair by HR by acting upstream of Rad51 recruitment to DSBs.

### Syk is required for DSB end resection activity

**Figure 3:**
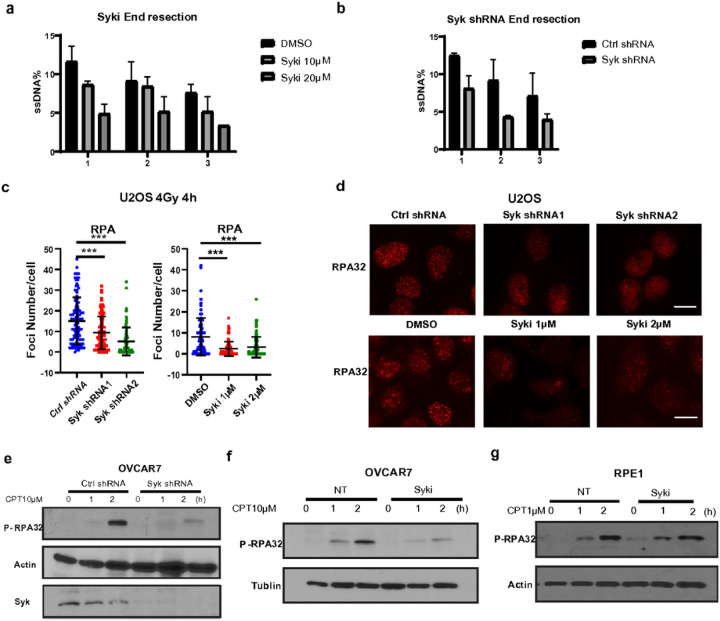
Syk is required for DSB end resection activity in Syk-expressing tumor cells. (a-b) ER-AsiSI U2OS cells were treated with Syk inhibitor, R406, at the indicated doses (a) or infected with lentiviruses expressing control (Ctrl) or Syk shRNA (b). The genomic DNA extracted from these cells was digested with BsrGI. DNA end resection adjacent to DNA double-strand break sites was then measured by qPCR. (c-d) U2OS cells were infected with Ctrl or Syk shRNAs and exposed to 4 Gy irradiation. Four hours later, cells were stained with RPA (Red) immunofluorescent antibody. (c) RPA foci number per cell in each condition was quantified. Representative data (mean±SEM) are shown from three independent experiments. n=50 cells. (d) Representative immunofluorescence images are shown. Scale bar, 20μm. (e) OVCAR 7 cells were infected with Ctrl or Syk shRNAs and then exposed to 10μM camptothecin (CPT) for the indicated times. Cells were then harvested and immunoblot was performed with the indicated antibodies. (f-g) OVCAR7 cells (f) and RPE1 cells (g) were treated with R406 (10μM) for one hour and then exposed to 10μM camptothecin for the indicated time. Cells were then harvested and immunoblot was performed with the indicated antibodies

To further localize where Syk may be acting in the HR pathway we examined the impact of Syk on end-resection, which is required for DSB repair to proceed by HR over NHEJ. Both Syk inhibition and Syk knockdown reduced the number of resections intermediates adjacent to DSBs in an ER-AsiSI end resection system (Fig. 3a–b,Extended Data Fig. 3c). Following resection, single stranded DNA is immediately coated by the replication protein A (RPA) complex^31^. Syk knockdown or Syk inhibition blocked RPA32 foci formation after IR, also potentially indicative of inhibited resection activity (Fig. 3c–d, Extended Data Fig. 3d).We also evaluated the impact of Syk on phosphorylated(p) RPA2 levels. As expected, treatment with the topoisomerase I inhibitor, CPT, which induces DSBs during replication, increased p-RPA2 expression in both OVCAR7 and RPE1 cells (Fig. 3e–g). Importantly, Syk knockdown and Syk inhibition blocked CPT-induced p-RPA2 levels in Syk-expressing OVCAR7 cells (Fig. 3e–f). However, Syk inhibition had no impact on p-RPA2 expression in Syk non-expressing RPE1 cells (Fig. 3g). These findings were consistent with our observations that targeting Syk specifically sensitized Syk-expressing tumor cells to DNA damaging therapy (Fig. 1g–m, Extended Data Fig. 2a-m). Therefore, we concluded that Syk activation may be necessary to carry out end resection and HR repair of both replicative and non-replicative induced DSBs specifically in Syk expressing cancer cells.

### Syk phosphorylates CtIP at T847 to promote end resection

**Figure 4:**
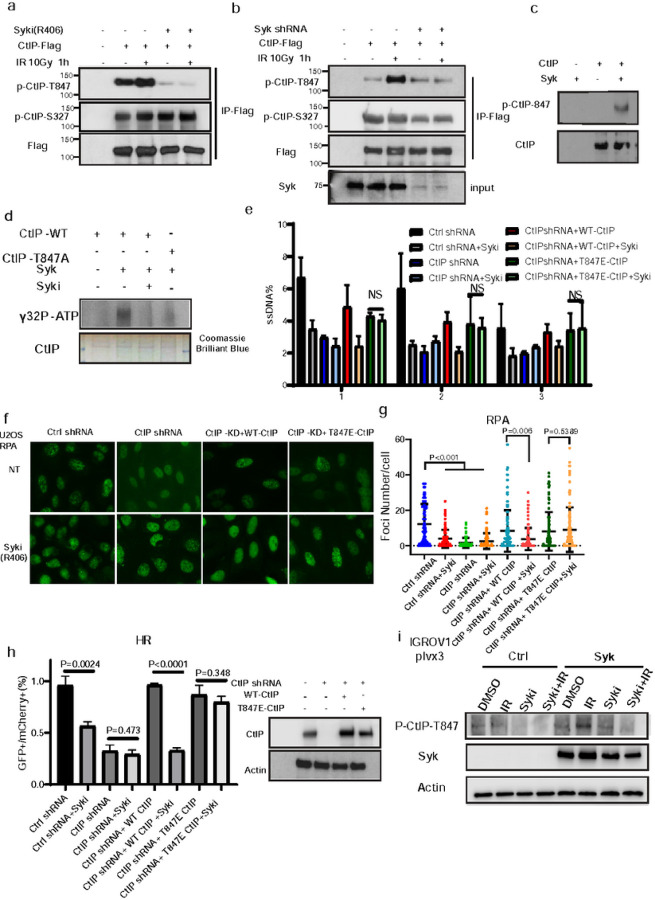
Syk phosphorylates CtIP at Thr847 to promote end resection. (a) CtIP-Flag constructs were expressed in 293T cells and those cells were treated with the Syk inhibitor, R406 (10μM). Cells were then exposed to 10 Gy IR and collected at the indicated timepoints. Immunoprecipitation with anti-Flag beads was performed followed by immunoblot with the indicated antibodies. (b) Endogenous Syk was knocked down in 293T cells using shRNA, and CtIP-Flag constructs were expressed. Cells were exposed to 10 Gy IR and collected at indicated timepoints. Immunoprecipitation with anti-Flag beads was performed followed by immunoblot with the indicated antibodies. (c-d) In vitro phosphorylation of Threonine 847 residue on CtIP peptide by purified recombinant Syk. 0.3 μg wildtype and mutant CtIP peptides were used as potential substrates in hot kinase assays along with 0.1 μg recombinant murine Syk in the presence of 1 mCi of [γ−32P] ATP. (c) Immunoblot with antibodies targeting CtIP and phosphorylate CtIP on the T847 residue were presented (d) Blot for [γ−32P] ATP and Coomassie brilliant blue. (e) Control or CtIP-depleted U2OS-Asisi cells were transfected with indicated constructs. 24 hours later, genomic DNA was extracted and digested with BsrGI. DNA end resection adjacent to DNA double-strand break sites was measured by qPCR. (f-g) Control or CtIP-depleted U2OS cells were transfected with indicated constructs for 24 h before treatment with 4 Gy IR for 2 h. RPA2 foci formation was then detected by immunofluorescence (f) and quantified (g). Data are representative of three independent experiments. Each dot represents a single cell, and 100 cells were counted in each group for this experiment. Error bars represent SEM from this experiment. Scale bar, 20μm. (h) Control or CtIP-depleted 293T cells were transfected with the indicated constructs. 24 hours later, cells were transfected with an HR reporter plasmid and HR was quantified, as previously described. Error bars represent SEM from three independent experiments. (i) Syk was overexpressed in 293T cells using Syk-plvx3. Cells were exposed to 10 Gy IR or R406 10μM and collected 1h later. Cells were then harvested and performed followed by immunoblot with the indicated antibodies

The recruitment of CtIP and BRCA1 to DNA damage sites is required for proficient DSB resection. Unlike RPA32 (Fig. 3c–d), CtIP and BRCA1 foci formation was not impacted by Syk knockdown or Syk inhibition (Extended Data Fig. 4a and b), suggesting that Syk may function in end-resection downstream of CtIP and BRCA1 recruitment to DSB sites. For CtIP-mediated end-resection and genome maintenance, both CtIP recruitment to DSBs and CtIP nuclease activity are required^32,33^. CtIP Ser327 phosphorylation is essential for CtIP recruitment whereas Thr847 phosphorylation is mainly required for CtIP resection activity^34^. Prior studies have suggested that Syk can function as a Ser/Thr kinase^35^. Thus, we examined the impact of Syk on CtIP-Ser327 and CtIP-Thr847 phosphorylation. CtIP-Ser327 phosphorylation was not impacted by Syk depletion of Syk inhibition in Syk expressing 293T cells (Fig. 4a–b). However, IR or cisplatin-induced phosphorylation of CtIP-Thr847 was abolished by either Syk knockdown or Syk inhibition (Fig. 4a–b, Extended Data Fig. 4c). Because Syk is a well-established tyrosine kinase, we also tested the possibility that CtIP tyrosine sites could be modulated by Syk using an anti-pTyr (G410) antibody which can detect total tyrosine phosphorylation levels. However, tyrosine phosphorylation was not impacted by IR, Syk knockdown, or Syk inhibition (Extended Data Fig. 4d-f). Based on these results, we hypothesized that Syk may promote DSB resection and HR following DNA damage through the phosphorylation of CtIP-T847.

To further explore this possibility, we examined phosphorylation of CtIP p-T847 in the presence of Syk and ATP using the *in vitro* kinase assay in control and Syk inhibitor treated conditions. ATP-dependent signal could be detected by an antibody recognizing p-T847 when human CtIP was co-incubated with active Syk kinase (Fig. 4c, Extended Data Fig. 4g), Moreover, p-T847 was blocked by Syk inhibition (Extended Data Fig. 4h). To further examine the specificity of Syk for CtIP-Thr847 phosphorylation we generated an 847 Threonine to Alanine (T847A) CtIP mutant and evaluated its ability to be phosphorylated in the presence of active Syk kinase and [γ−32P] ATP (Fig. 4d). We found that WT but not the T847A CtIP mutant could be phosphorylated, and that WT CtIP-Thr847 phosphorylation could be abolished by Syk inhibition (Fig. 4d). These findings suggested that CtIP-Thr847 is a substrate of Syk.

Previous reports have suggested that mutation of threonine 847 on CtIP to glutamic acid will mimic T847 phosphorylation and constitutively activate CtIP end resection activity^34^. To further test the hypothesis that Syk specifically regulates DSB resection and HR through CtIP T847 we knocked down CtIP and reconstituted these cells with wild type CtIP or T847E CtIP. In contrast to our observations of Syki abrogating DSB resection in WT CtIP expressing cells (Fig. 3a–c), Syk inhibition was unable to block the formation of ssDNA resection intermediates adjacent to DSBs (Fig. 4e) or RPA foci (Fig. 4f–g) in cells expressing T847E mutant CtIP. In addition, Syk inhibition could not abrogater HR activity in constitutively activated T847E mutant CtIP expressing cells (Fig. 4h). Moreover, Syk overexpression in IGROV1 parental cells could further enhance T847 phosphorylation on CtIP(Fig. 4i). Neither Syk knockdown nor Syk inhibition affected cell cycle progression (Extended Data Fig. 5a, b), excluding the possibility that Syk may be regulating CtIP Thr 847 by inhibiting cell cycle progression. Thus, we concluded from these experiments that Syk inhibition abrogates the phosphorylation of the Thr847 site of CtIP to inhibit end resection and HR.

### Syk is phosphorylated by ATM following DNA damage

**Figure 5:**
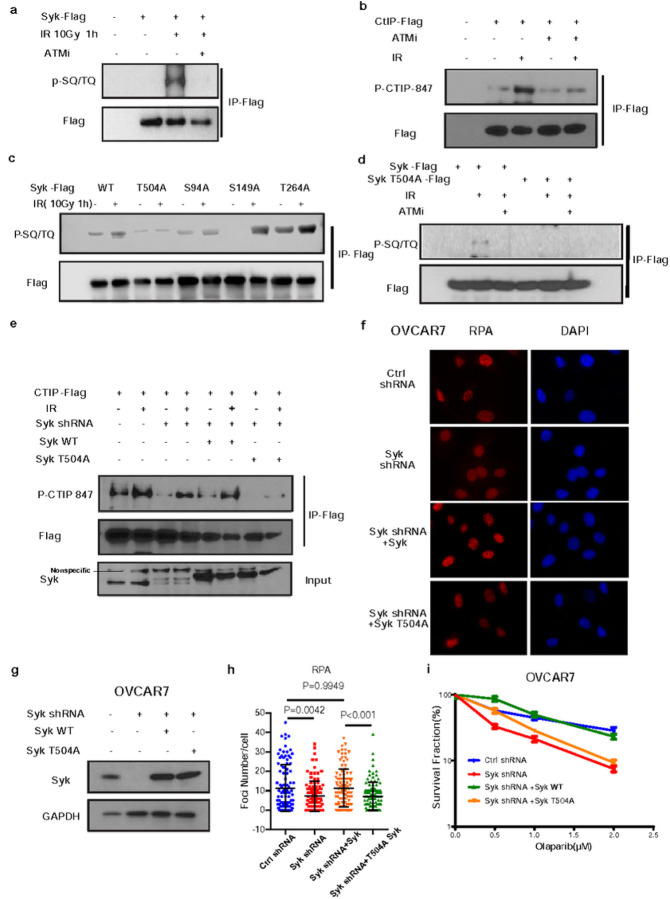
Syk is phosphorylated by ATM following DNA damage. (a-b) 293T cells transfected with FLAG tagged Syk were treated with DMSO or ATM inhibitor AZD0156 (100nM) for 2 h prior to 10 Gy IR. One hour later cells were harvested cells and immunoprecipitated with anti-FLAG agarose beads. After treatment with protein phosphatase, blots were probed with indicated antibodies. (c-d) 293T cells transfected with FLAG tagged wild-type Syk or indicated mutant Syk were treated with DMSO or AZD0156 (100nM) for 2 h prior to IR. Harvested cells were immunoprecipitated with anti-FLAG agarose beads and blots were probed with the indicated antibodies. (e) CtIP-Flag, wild type or mutant Syk constructs were expressed in wild type or Syk deleted 293T cells. Cells were then exposed to 10 Gy IR and immunoprecipitation was performed 1 hour later with anti-Flag beads. Blots were probed with the indicated antibodies. (f-g) Syk-depleted OVCAR7 cells were transfected with wild-type or the indicated mutant Syk before treatment with 4 Gy IR. 4 hours later, RPA2 foci formation was detected by immunofluorescence (f) and quantified (g). Data are representative of three independent experiments. Each dot represents a single cell. Error bars represent SEM from three independent experiments. Scale bar, 20 μm. (h) Endogenous Syk was knocked down in OVCAR7 cells. Cells were then transfected with the indicated Syk WT or SykT504A plasmids and immunoblot was performed with the indicated antibodies. (i) Cells from h were exposed to the indicated doses of olaparib and clonogenic survival was assessed. Error bars represent SEM from three independent experiments

Since Syk-mediated phosphorylation of CtIP was induced by DNA damage, this raised the possibility that Syk itself may be post-translationally modified by the DNA damage response signalling pathway. ATM is a key upstream DNA damage response kinase that orchestrates the cellular response to DSBs and replication stress. We found that Syk was phosphorylated at SQ/TQ motifs, which are ATM/ATR consensus phosphorylation sites, following IR (Fig. 5a) and cisplatin (Extended Data Fig. 6a). In addition, Syk SQ/TQ site phosphorylation was blocked by ATM inhibition (Fig. 5a and Extended Data Fig. 6a). Moreover, phosphorylation of CtIP pT847, which we established to be a Syk substrate(Fig. 4), was abrogated by ATM inhibitor treatment (Fig. 5b). Collectively, these findings suggested that ATM may regulate Syk activity following DNA damage.

We next analyzed the sequence of Syk for candidate SQ/TQ sites that may be phosphorylated following DNA damage(Fig. 5c–d). We found that mutation of threonine 504 on the C-terminal tyrosine kinase domain of Syk, to alanine (T504A) but not other Syk SQ/TQ sites, abolished Syk SQ/TQ motif DNA damage-induced phosphorylation (Fig. 5c–d). Based on this data, we hypothesized that phosphorylation of Syk T504 may promote CtIP-mediated DSB resection and HR activity.

To test our hypothesis and gain an improved understanding of the potential importance of Syk T504 on the regulation of CtIP and DNA DSB repair we reconstituted wild type Syk or T540A Syk into 293T cells in which endogenous Syk was knocked down (Fig. 5e), and examined CtIP phosphorylation after DNA damage. As displayed in Fig. 5e, CtIP pT847 could be rescued by expression of Syk WT, but not the Syk T504A mutant. In addition, Syk T504A mutation abolished RPA foci formation (Fig. 5f–h). Further, while Syk knockdown OVCAR7 cells were hypersensitive to PARP inhibition (Extended Data Fig. 2a), only expression of WT Syk but not the Syk T504A mutant in Syk knockdown cells could restore PARP inhibitor resistance (Fig. 5i). Taken together, these data suggest that Syk T504 phosphorylation is induced by ATM and is essential for CtIP T847 phosphorylation, end resection and HR activity in Syk expressing tumor cells.

### ATM promotes Syk recruitment to DNA DSB sites through NBS1

**Figure 6:**
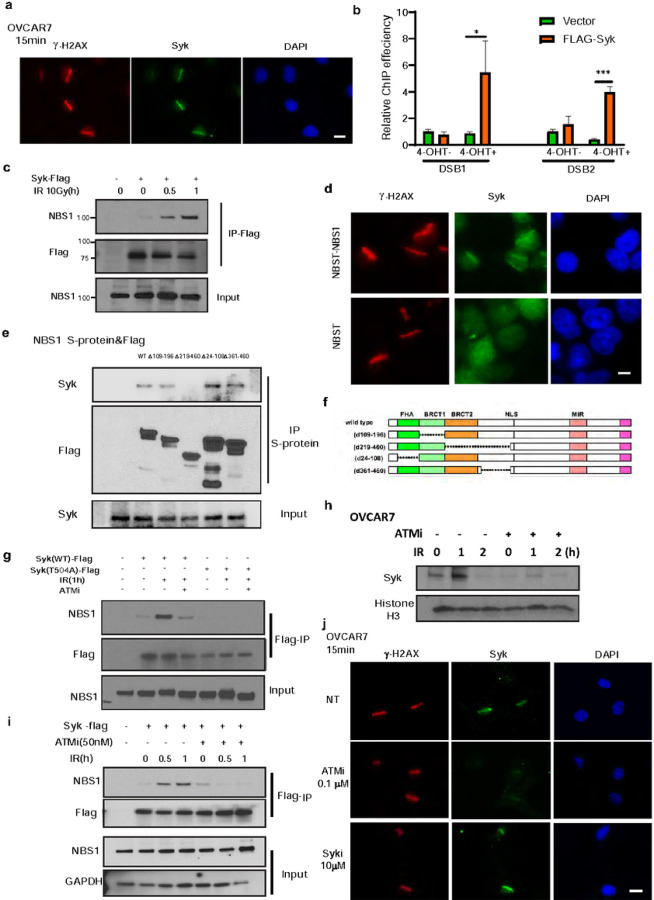
Syk is activated by ATM and recruited to DNA DSB sites by NBS1 (a) OVCAR7 cells were stained with Syk(Green) and γH2AX(Red) 15 minutes after microirradiation. Scale bars, 20 μm. (b) ER-AsiSI U2OS cells transfected with FLAG–Syk were added with or without 4-OHT. ChIP assay was then performed using FLAG antibody. (c) Syk-Flag constructs were expressed in 293T cells. 24 hours later, cells were exposed to 10 Gy IR and collected at indicated timepoints. Immunoprecipitation with anti-Flag beads was performed. Blots were probed with the indicated antibodies. (d) NBS1 deficient cells, NBST, and NBS1 proficient cells, NBST-NBS1, were exposed to microirradiation. 15 minutes later cells were stained with Syk (Green) or γH2AX (Red) immunofluorescent antibodies. Scale bars, 10 μm. (e-f) Indicated NBS1-truncation constructs (f) with S-protein tag were expressed in 293T cells (e). 24 hours later, cells were exposed to 10 Gy IR. After one hour, cells were collected and immunoprecipitation with anti-Flag beads was performed followed by immunoblot with the indicated antibodies (e). (g-i) Syk-Flag and Syk T504A constructs were expressed in 293T cells. 24 hours later, cells were treated with 50nM ATMi and exposed to 10 Gy IR and collected at indicated timepoints. Immunoprecipitation with anti-Flag beads was performed. Blots were probed with the indicated antibodies. (h) OVCAR7 cells were treated with 10 Gy IR and then harvested at the indicated time points. The chromatin binding proteins were extracted and subjected to immunoblot with the indicated antibodies. (j) OVCAR7 cells were treated with ATMi (AZD0156) or Syki (R406) at the indicated doses and stained with Syk (Green) or γH2AX (Red) immunofluorescent antibodies 15min after micro-irradiation. Scale bars, 20 μm

Syk is reported to be found in both the nuclear and cytoplasmic cellular compartments. Therefore, we examined the impact of DNA damage on the cellular localization of Syk in OVCAR 7 cells. Syk co-localized with γ-H2AX following high dense UV treatment (Fig. 6a). In addition, Syk was recruited to site-specific DSBs in ER-AsiSI U2OS cells, as detected by ChIP analysis (Fig. 6b). These results suggested that Syk is recruited to DNA DSBs in response to DNA damage. We next investigated potential DNA damage response elements that might regulate Syk recruitment to DSBs. The MRN complex, consisting of Mre11, Rad50 and Nbs1, binds avidly to DSBs to initiate end resection and DSB repair. Using immunoprecipitation, we found that Syk interacted with NBS1, but not BRCA1,RPA2,RAD51 or MRE11,and that the interaction between Syk and NBS1 increased post IR (Fig. 6c, Extended Data Fig. 6b).

**Figure 7:**
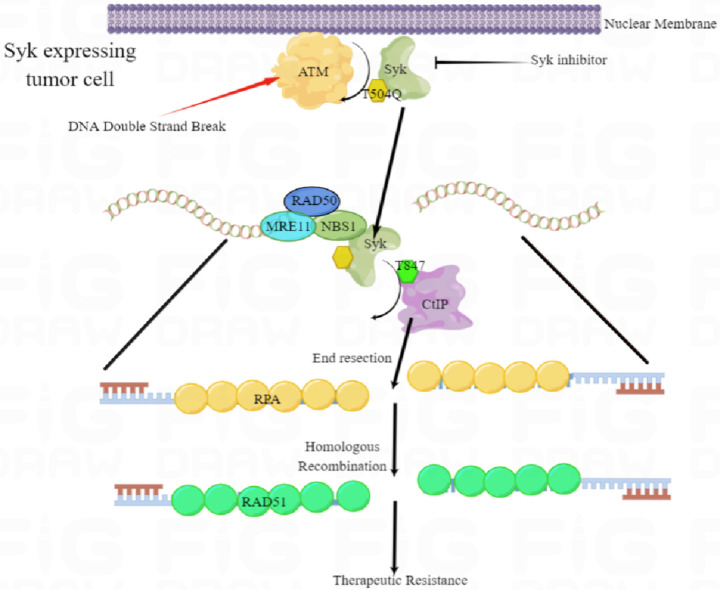
Schema displaying working model of the role of Syk in regulating HR in Syk expressing therapeutically resistant TNBC and HGSOC. ATM phosphorylates T504Q on Syk which promotes Syk’s interaction with NBS1 and Syk recruitment to double strand breaks. Once at the site of DNA damage, Syk phosphorylates CtIP Thr 847 to promote DNA end-resection, HR, and resistance to DNA targeted therapy

Based on this data, we hypothesized that the Syk-NBS1 interaction may be required for Syk recruitment to DSBs. Indeed, Syk localization at sites of UV damage was abolished in NBS1 KO cell lines (Fig. 6d). To further test our hypothesis and examine how Syk and NBS1 interact we performed NBS immunoprecipitation following expression of wild-type or various NBS1 truncations in 293T cells. Our findings suggested that the BRCT2 domain of NBS1 may be most important for the NBS1 interaction with Syk (Fig. 6e–f). Given our data suggesting that Syk T504 phosphorylation is induced by ATM and promotes Syk mediated end resection and HR (Fig. 5c–i),we hypothesized that ATM-mediated phosphorylation of Syk T504 might also be required for the NBS1-Syk interaction and Syk’s recruitment to DSBs. Indeed, the Syk T504A mutation blocked the Syk-NBS1 interaction (Fig. 6g). Moreover, ATM inhibition reduced the nuclear accumulation of Syk following IR (Fig. 6h) and abrogated the interaction of Syk with NBS1 (Fig. 6i). Interestingly, treatment with an ATM inhibitor, but not a Syk inhibitor, abolished Syk recruitment to UV laser damage sites, suggesting that phosphorylation of Syk at T504 by ATM, but not Syk kinase activity, may be necessary for Syk recruitment to damage sites (Fig. 6j). Thus, we propose a new model by which Syk promotes resistance to DNA targeted therapy through an alternative HR activation pathway (Fig. 7). In summary, ATM-mediated phosphorylation of Syk at T504 after DNA damage promotes Syk’s interaction with the BRCT2 domain of NBS1, and Syk recruitment to DNA DSBs, in Syk expressing tumor cells. Once at DSBs, Syk phosphorylates CtIP T847 to promote end resection activity, HR, and therapeutic resistance (Fig. 7).

## Discussion

In this paper, we discovered a new and unexpected function of Syk kinase in the DNA damage response pathway. Syk is best known for mediating signal transduction in immune cells downstream of transmembrane receptors. Here, we linked Syk with several key DNA damage response pathway elements including ATM, NBS1 and CtIP in Syk-expressing TNBC and HGSOC cells. By post-translationally modifying CtIP, we found that Syk promotes DSB resection and repair by HR specifically in Syk expressing tumor cells. Further, by promoting DSB resection and HR, expression of Syk may be a mechanism of acquired resistance to DNA damaging cisplatin and PARP inhibitor treatment. Our results suggest that pharmacologic inhibition of Syk, which is already an approved clinically available therapy for idiopathic thrombocytopenia, may be a promising strategy for clinical investigation in combination with DNA targeted therapy in Syk expressing tumors. Further, our results indicate that Syk protein expression in tumors could serve as a potential biomarker of response.

Typically protein kinases discriminate between serine/threonine and tyrosine depending on their structures^36^. Tyrosine kinase activity of Syk has been most studied. However, Syk has also been previously reported to phosphorylate several Serine substrates. For example, Serine 197 (S197) in the cytoplasmic tail of Ig-α n can be phosphorylated upon B-cell antigen receptor activation by Syk^35^. In addition, Syk regulates the G2 checkpoint by physically associating with and inhibiting the dual-specificity phosphatase CDC25C via phosphorylation of its S216 residue^37^. Further, Syk phosphorylates Ikaros at unique C-terminal serine phosphorylation sites S358 and S361, thereby augmenting Ikaros nuclear localization and sequence-specific DNA binding activity^38^. Here, we found Threonine 847(T847) on CtIP can also be phosphorylated by Syk *in vitro* and *in vivo*, thereby promoting CtIP DSB resection activity. Thus, Syk is a multi-function kinase that phosphorylates tyrosine, serine and threonine sites^39–41^.

Of note, PARP inhibition, radiotherapy, and other DNA targeted therapies can have both immunosuppressive and immune stimulating effects, including through activations of the DNA-sensing cGAS-STING signaling pathway^42,43^. Syk inhibition has also previously been reported reported to have potential immune stimulatory effects in solid tumors which are context dependent, including by promoting macrophage polarization towards more immune stimulatory macrophage populations^44^. Further investigation is needed to characterize the effects of the combination of Syk inhibition and DNA targeted therapy on the tumor microenvironment in immune competent Syk expressing HGSOC and TNBC models.

In summary, we have identified a novel role of Syk in promoting DNA repair and therapeutic resistance. Following DNA damage in Syk expressing cells, Syk is activated by ATM and recruited to DNA double strand breaks by NBS1, where Syk phosphorylates CtIP at Thr-847 to promote resection and HR. Moreover, Syk inhibition induces HR deficiency and sensitizes tumor cells to DNA targeted therapy. Investigation of Syk inhibition as a tumor-specific strategy to induce HR deficiency and enhance the therapeutic ratio of DNA targeted therapy in Syk expressing tumors is warranted.

## Materials and Methods

### Cell culture and transfection.

Ovcar7, Ovcar8, Ovcar10, Ovcar90, Ovcar56, A2780, HEK293T, U2OS and TERT-RPE1 cell lines were all purchased from ATCC. EFO-27 cells were purchased from DSMZ company. Human ovarian epithelial cells (HOSE) were purchased from ScienCell company. Cells were grown in DMEM, McCoy’s 5A or RPM1640 which mixed with 10% FBS. Transfection reagent is TransIT-X2 from Mirus Bio LLC.

### shRNA.

Syk shRNA(NM_003177)and CtIP(NM_002894) shRNA were purchased from Sigma-Aldrich. The lentivirus was made according to the company’s protocol.

Syk shRNA no.1 5′-GCGCAATTACTACTATGACGT-3′

Syk shRNA no.2 5′-GCATGAGTGATGGGCTTTATT-3′

CtIP shRNA: 5′-CGGCAGCAGAATCTTAAATT-3′

### HR/NHEJ assay

Cells expressing indicated shRNAs or constructs were co-transfected with DR-GFP/NHEJ, pCBA-I-SceI, and m-Cherry using TransIT-X2. cells were harvested and analyzed by fluorescence-activated flow cytometry (FACS) to examine the percentage of GFP-positive cells 36–48h later. Results were normalized to control group and m-Cherry was employed to normalize for transfection efficiency. The FACS sequential gating/sorting strategies was described previously (site our lab paper).

### Plasmids, reagents and antibodies.

pWZL Neo Myr Flag Syk was purchased from addgene and subcloned into plvx3 vector. GFP-CtIP were generously provided by Dr. Junjie Chen (MD Anderson Cancer Center, TX) and subcloned into plvx3. Flag–CtIP–T847E was purchased from Addgene. Anti-FLAG agarose, 3×FLAG peptide and ATM inhibitor KU55933 were purchased from Sigma Aldrich. Olaparib was purchased from LC-lab. Syk inhibitor R406 was purchased from selleckchem.

Syk(D3Z1E, 13198) antibody, anti-SQ/TQ motif (6966) and anti-pS345 Chk1 (2348) was purchased from CST. anti-CtIP (Thr847) (p1012–847, 1:2000), and anti-CtIP (Thr327) (p1012–327, 1:2000) were purchased from PhosphoSolutions. anti-RPA32 (sc-56770) and anti-CtIP (sc-271339 for WB) were purchased from Santa Cruz; anti-FLAG (F1804) was purchased from Sigma; anti-CtIP (61141, 1:1000 for IF) was purchased from Active Motif; anti- γ-H2AX antibody(05–636) was purchase from Millipore. Anti-GAPDH (60004–1-lg) was purchased from Proteintech; anti-Rad51 (GTX100469 for IF) was purchased from Genetex. Anti-H3(17168–1, AP) was purchased from Proteintech. All the secondary antibody were purchased from Jackson ImmunoResearch.

### Immunofluorescence staining

Immunofluorescence staining was performed according to standard process. Briefly, Ovcar7 or U2OS cells were seeded and transfected with indicated plasmids in six-well plates containing coverslips. For most antibody staining after treatment with the appropriate dose of irradiation or inhibitors, cells were fixed at the indicated time points using 4% paraformaldehyde for 20 min, washed three times in 1 × PBS, and then extracted with 0.5% Triton X-100 PBS solution for 5–10 min. For RPA2 foci staining, cells were fixed with methanol: acetone (1:1) at − 20°C for 30 min. Then cells were incubated with the indicated primary antibodies overnight, washed three times with 1 × PBS and incubated with Alexa Fluor 488- or Alexa Fluor 594-conjugated secondary antibody for 30 min at room temperature. Finally, cells were stained with 100 ng/ml 4, 6-diamidino-2-phenylindole (DAPI) for 3–5 min to visualize nuclear DNA. The coverslips were mounted onto glass slides using anti-fade solution. Finally, the slides were visualized using Leica ECLIPSE E800 fluorescence microscope with a 40× objective lens (NA 1.30). Foci was quantified using Image J software.

### Colony-formation assay

Ovcar7, U2OS, IGROV1 or IGROV1 Cisplatin resistance cells (500–3000) were seeded in triplicate in each well of six-well plates. After 8–16 h, cells were treated with indicated inhibitors or exposed to ionizing radiation (IR) at indicated doses. Plates were left for 7–16 days in the 37°C incubator to allow for colony formation. Colonies were fixed with methanol, stained with 5% GIEMSA for 10 min, and then counted. The results were normalized to the plating efficiencies of the untreated group.

### Western blot and Co-immunoprecipitation

#### Western blot and Co-immunoprecipitation

Cells were harvested and lysed with NETN buffer (20 mM Tris–HCl, pH 8.0, 1 mM EDTA, 100 mM NaCl, and 0.5% NP-40) containing protease inhibitors on ice for 30 min. After centrifugation at 12,000 × g for 15 min, supernatant containing proteins was immunoprecipitated by incubating with indicated antibodies or agarose beads overnight at 4°C. The immunoprecipitates were washed with NETN buffer and then centrifuged at 800 × g for 1 min for three times, boiled in 1× SDS loading buffer for 5 min, and separated on sodium dodecyl sulfate polyacrylamide gel electrophoresis (SDS-PAGE) and blocked in 5% milk TBST buffer, and then detected with antibodies as indicated. Uncropped and unprocessed scans of the most important blots now can be found in Extended Data figure in the Extended Data Information.

### Tumor xenograft

All experiments were performed with the approval of the Institutional Animal Care and Use Committee at Mayo Clinic (Rochester, MN). All mice used in this study were maintained under specific pathogen-free conditions, 21 ± 2°C relative humidity of 45 ± 15%, and a 12-h light/dark cycle. IGROV1 cisplatin resistance cells were subcutaneously injected into the flanks of 6-week-old female athymic nude Ncr nu/nu (NCI/NIH) mice using 18-gauge needles. Each mouse received injections of a 0.2-ml mixture of 2 million cells with 50% growth factor-reduced MATRIGEL (BD Bioscience). Mice bearing tumors of 50 mm^3^ were randomly assigned into the indicated groups: vehicle control (saline), PARPi (purchased from LC-lab, O-9201, 50 mg/kg), Fostamatinib (purchased from Selleck Chemicals, 80mg/kg). The treated mice were intraperitoneally injected with PARP inhibitor and gavaged with Fostamatinib 5 times/week. Tumor volume was measured every 7 days using calipers, and tumor volume was calculated using the formula length × width^2^. Mice were sacrificed for tumor dissection on day 24 of treatment.

### DNA resection measurement

The percentage of ssDNA (ssDNA%) generated by resection was determined as previously described54. Briefly, ER-AsiSI U2OS cells expressing indicated shRNAs or constructs were treated with 1 μM 4-OHT for 4 h, cells were then harvested and genomic DNA was extracted with DNAzol reagent (Invitrogen) according to manufacturer’s instruction. After that, 500 ng genomic DNA sample was digested or mock digested with BsrGI enzyme at 37°C overnight. 2 μL DNA were used as templates in 25 μl of qPCR reaction containing 12.5 ml of 2× Taqman Universal PCR Master Mix (ABI), 0.5 mM of each primer and 0.2 mM probe. The sequences of qPCR primers and probes are shown in Extended Data Table 1. ΔCt was calculated from the Ct value of the digested sample subtracting the mock-digested sample. The ssDNA% was calculated with the following equation: ssDNA% = 1/(2(△Ct − 1) + 0.5)*100.

### Statistics and reproducibility

For cell survival assay, data are presented as the mean ± S.E.M. of three independent experiments. All the Western blotting and micrograph data were repeated independently three times with similar results. For the animal xenograft studies, tumor volume data are presented as the mean ± S.E.M. with seven mice per arm. Statistical analyses were performed in Microsoft Excel, GraphPad Prism7 with ANOVA, the Student’s t test, or χ2 test. Statistical significance is represented in figures by *p < 0.05; **p < 0.01, ***p < 0.001, n.s., not significant. The flow cytometry data were gathered by Attune NxT Flow Cytometer software v2.6 and analyzed by flowjo.

## Data Availability

All correspondence and material requests should be addressed to R.W.M and Z.L.
